# Histone Acetylation of Immune Regulatory Genes in Human Placenta in Association with Maternal Intake of Olive Oil and Fish Consumption

**DOI:** 10.3390/ijms20051060

**Published:** 2019-03-01

**Authors:** Nathalie Acevedo, Paolo Frumento, Hani Harb, Bilal Alashkar Alhamwe, Catharina Johansson, Lisa Eick, Johan Alm, Harald Renz, Annika Scheynius, Daniel P. Potaczek

**Affiliations:** 1 Department of Clinical Science and Education, Karolinska Institutet, and Sachs’ Children and Youth Hospital, Södersjukhuset, 11883 Stockholm, Sweden; nacevedoc@unicartagena.edu.co (N.A.); catharina.johansson@ki.se (C.J.); johan.alm@ki.se (J.A.); annika.scheynius@ki.se (A.S.); 2 Institute for Immunological Research, University of Cartagena, Cartagena 130015, Colombia; 3 Unit of Biostatistics, Institute of Environmental Medicine, Karolinska Institutet, 17177 Stockholm, Sweden; paolo.frumento@ki.se; 4 Institute of Laboratory Medicine, member of the German Center for Lung Research (DZL), Universities of Giessen and Marburg Lung Center (UGMLC), and the inVIVO Planetary Health, Group of the Worldwide Universities Network (WUN), Philipps-University Marburg, 35043 Marburg, Germany; hani.harb@childrens.harvard.edu (H.H.); alashkab@staff.uni-marburg.de (B.A.A.); lisaeick@t-online.de (L.E.); harald.renz@uk-gm.de (H.R.); 5 Division of Immunology, Boston Children’s Hospital, Harvard Medical School, Boston, MA 02115, USA; 6 College of Pharmacy, International University for Science and Technology (IUST), Daraa 15, Syria; 7 Clinical Genomics, Science for Life Laboratory, 17165 Stockholm, Sweden; 8 John Paul II Hospital, 31-202 Krakow, Poland

**Keywords:** ALADDIN, fish, H3, H4, histone acetylation, immune genes, maternal diet, olive oil, placenta, pregnancy

## Abstract

Maternal diet modifies epigenetic programming in offspring, a potentially critical factor in the immune dysregulation of modern societies. We previously found that prenatal fish oil supplementation affects neonatal T-cell histone acetylation of genes implicated in adaptive immunity including *PRKCZ*, *IL13*, and *TBX21*. In this study, we measured H3 and H4 histone acetylation levels by chromatin immunoprecipitation in 173 term placentas collected in the prospective birth cohort, ALADDIN, in which information on lifestyle and diet is thoroughly recorded. In anthroposophic families, regular olive oil usage during pregnancy was associated with increased H3 acetylation at *FOXP3* (*p* = 0.004), *IL10RA* (*p* = 0.008), and *IL7R* (*p* = 0.007) promoters, which remained significant after adjustment by offspring gender. Furthermore, maternal fish consumption was associated with increased H4 acetylation at the *CD14* gene in placentas of female offspring (*p* = 0.009). In conclusion, prenatal olive oil intake can affect placental histone acetylation in immune regulatory genes, confirming previously observed pro-acetylation effects of olive oil polyphenols. The association with fish consumption may implicate ω-3 polyunsaturated fatty acids present in fish oil. Altered histone acetylation in placentas from mothers who regularly include fish or olive oil in their diets could influence immune priming in the newborn.

## 1. Introduction

Maternal diet can modify the epigenetic landscape in diverse tissues and is a crucial factor in the developmental programing of the offspring [1,2,3]. Human and mice studies support the substantial effects of maternal diet on the expression of metabolic, immune, and neurodevelopmental genes, which is mediated in part through the alteration of histone acetylation marks [4,5,6]. Histone acetylation is an epigenetic mechanism that consists in the addition of acetyl groups to lysines protruding in the N-terminal tail of histones, a process mediated by histone acetyltransferases, histone deacetylases, and complex epigenetic machineries. Increased acetylation of histones H3 and H4 is typically associated with open chromatin leading to better accessibility of promoters to the transcriptional machinery and hence higher gene expression [7,8].

Several compounds (i.e., sulforaphane, curcumin, polyphenol flavonoids, butyrate, indole-3-carbinol, and organosulfur compounds) have been shown to modify histone acetylation levels [9]. Recent experimental evidence highlights the role of fish oil and its bioactive component n-3 polyunsaturated fatty acids (PUFA) as significant epigenetic modifiers of histone marks [10]. The availability of n-3 PUFA during gestation modifies H3 acetylation in fetal neurons and astrocytes [5]. Moreover, we recently found that fish oil supplementation affects histone acetylation levels in neonatal T cells, affecting immune genes such as protein kinase C zeta gene (*PRKCZ*), *IL13*, and T-box 21 gene (*TBX21*) [11,12]. In addition, the gene forkhead box P3 (*FOXP3*), which encodes a master regulator of immune homeostasis, has shown to be epigenetically modified by dietary compounds including all-*trans* retinoid acid [13], microbial-derived butyrate [14], and propionate [15]. This gene is especially important because animal models suggest that *FOXP3* is epigenetically modified by dietary exposures, such as high fiber and acetate, during the prenatal period [16].

Placenta constitutes a critical interface mediating the intrauterine maternal effects on epigenetic programming [17]. For instance, maternal obesity is associated with altered mRNA expression of key acetylation modifiers in placenta, including histone deacetylases and lysine acetyltransferases [18]. In rats, dietary unsaturated fatty acids in maternal diet [eicosapentaenoic acid (EPA, 20:(5n−3)) + docosahexaenoic acid (DHA, 22:(6n−3))] exhibited a profound effect in modulating the epigenetic parameters in placenta and fetal liver [19]. Previous studies in the Assessment of Lifestyle and Allergic Disease During INfancy (ALADDIN) cohort suggested that CD14, a co-receptor of toll-like receptors critical in innate immune sensing, is epigenetically modified in human placenta, but the exposures involved require better definition [20]. In the present study, we hypothesized that acetylation levels of H3 or H4 histones at the promoters of immune regulatory genes are associated with lifestyle and diet during pregnancy. We evaluated histone acetylation in 173 placentas from the prospective birth cohort ALADDIN in which extensive information on lifestyle and diet has been previously recorded [21] in anthroposophic, partly anthroposophic, and non-anthroposophic families from the Stockholm area, Sweden. The dietary patterns related with the anthroposophic lifestyle in this cohort included an increased frequency of vegetarian diet, consumption of organic/biodynamic food, use of butter on bread, and use of olive oil as main cooking fat [21].

## 2. Results and Discussion

A total of 173 placenta specimens were analyzed by chromatin immunoprecipitation (ChIP) and quantitative polymerase chain reaction (PCR) to measure both H3 and H4 pan-acetylation levels at the promoters of four candidate immune genes, which have previously shown to be epigenetically modified by early life exposures (*CD14* and *FOXP3*) [16,20,22] or epigenetically altered in T cells (*IL10RA* and *ILR7*) [Acevedo et al., Unpublished]). The ALADDIN study includes families with anthroposophic, partly anthroposophic, and non-anthroposophic lifestyles [21]. As a result of the limited number of available placenta specimens in the anthroposophic group, in this study, this group was combined with the partly anthroposophic group (A + PA). The demographic characteristics of the participating families are presented in Table 1. Maternal age at birth, parity, offspring gender, and birth weight did not differ between the two groups.

**Figure 1:**
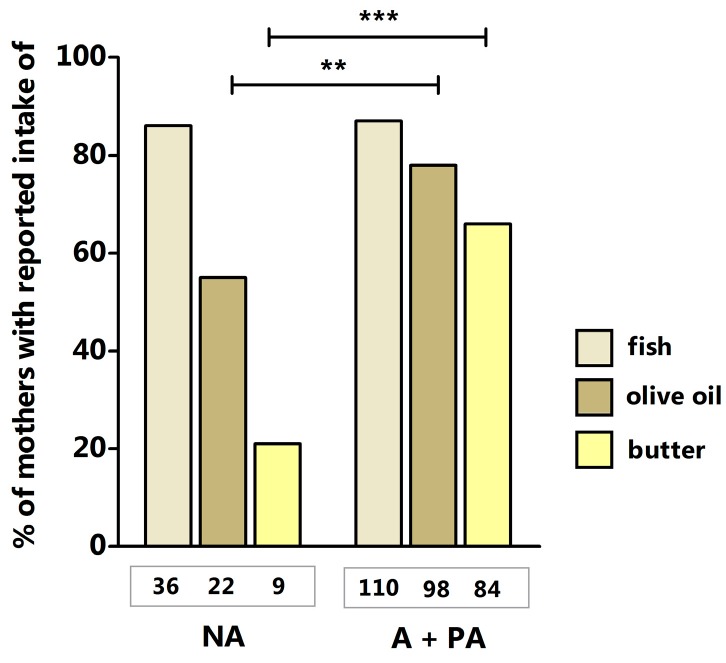
Prevalence of prenatal consumption of fish, olive oil, and butter in non-anthroposophic (NA), anthroposophic, or partially anthroposophic mothers (A + PA). Numbers of mothers with complete data on dietary exposures are presented below the bars. Fisher exact test: ** *p* = 0.007; *** *p* < 0.001.

In comparison with mothers from non-anthroposophic families, mothers with A + PA lifestyle reported a more frequent use of olive oil as main cooking fat during pregnancy [55% (22/40) vs. 78% (98/125), respectively; *p* = 0.007] and a more frequent consumption of butter or butter-based, full-fat margarine on bread during pregnancy [21% (9/42) vs. 66% (84/127), respectively; *p* < 0.001] (Figure 1). Maternal fish consumption during pregnancy did not differ between the two lifestyle groups (Figure 1). The distribution of these variables in this dataset clearly resembles that previously described by Stenius et al. [21].

**Figure 2:**
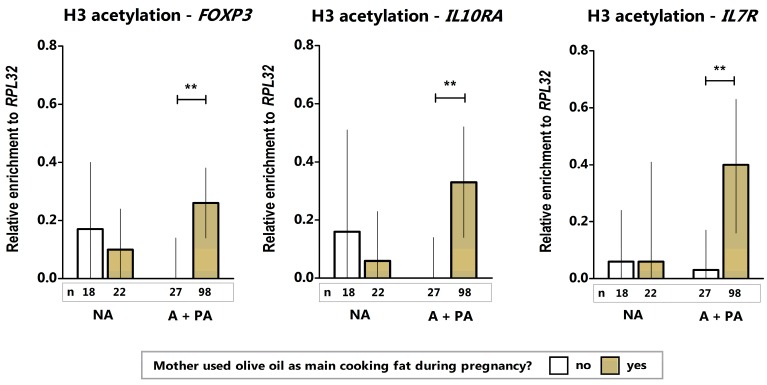
H3 histone acetylation levels (median with 95% confidence interval) in placenta according to the maternal use of olive oil as main cooking fat during pregnancy. NA denotes non-anthroposophic group; A, anthroposophic group; PA, partially anthroposophic group. Numbers of mothers with complete data on dietary exposures are presented below the bars. ** *p* value < 0.01.

We found that H3 acetylation levels at the promoters of *FOXP3*, interleukin 10 receptor subunit alpha (*IL10RA*), and interleukin 7 receptor (*IL7R*) genes were significantly increased in the placenta specimens from mothers who regularly used olive oil as the main cooking fat during pregnancy. This association was detected in mothers with an A + PA lifestyle but was not observed in non-anthroposophic mothers (Figure 2). H3 acetylation levels in placentas were not associated with maternal fish consumption during pregnancy or regular intake of butter or butter-based, full-fat margarine on bread (data not shown).

Demographic variables that could potentially influence placenta acetylation, such as maternal age at birth, parity, birth weight gestational age, or parental smoking (Table 1), were not associated with H3 acetylation levels of *FOXP3*, *IL10RA* or *IL7R* (data not shown). We then implemented median regression adjusting by offspring gender in the group of A + PA mothers (Table 2) to further confirm the association between maternal use of olive oil as main cooking fat during pregnancy on placenta histone acetylation.

These findings suggest that compounds in olive oil may have particularly important effects on the histone marks in placenta. Our results are in line with those showing that olive oil polyphenols (i.e., oleuropein aglycone) increase H3 and H4 histone acetylation by down regulating HDAC2 [23], and with the potential pro-acetylation effects of hydroxytyrosol (HT), a compound in *Olea europaea*, which can bind with high affinity to chromatin-modifying enzymes and inhibit HDAC6 and the lysine-specific histone demethylase 1 (LSD1) [24]. Because increased acetylation may lead to elevated transcriptional activities of these genes, we anticipated that a reduction in acetylation in placentas from mothers who do not include olive oil in their diets could modify the accessibility of *FOXP3*, *IL10RA*, and *IL7R* promoters to the transcriptional machinery and, accordingly, expression. This may affect early innate immune responses, anti-inflammatory mechanisms [25,26], generation of regulatory T-cells, and tolerance [27]. The association between olive oil intake and increased acetylation of *FOXP3* is of great interest because this gene is critical for immune regulation and has been found to undergo modification by polyunsaturated fatty acids during pregnancy [16].

**Figure 3:**
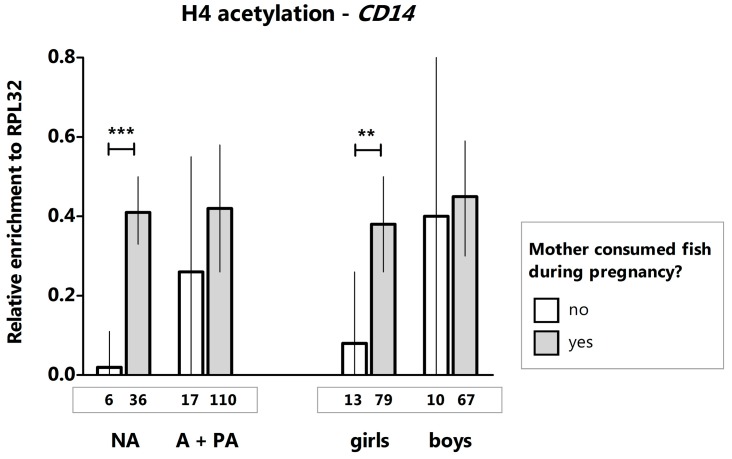
H4 histone acetylation levels (median with 95% confidence interval) at the *CD14* promoter in placenta according to maternal fish consumption during pregnancy. NA denotes non-anthroposophic group; A, anthroposophic group; PA, partially anthroposophic group. Numbers of mothers with complete data on dietary exposures are presented below the bars. *** *p* < 0.0001, ** *p* < 0.01.

Moreover, maternal fish consumption during pregnancy was associated with increased H4 histone acetylation at the *CD14* promoter in placentas of female offspring but not in males (Figure 3). This observation remained significant in either the crude median regression model [β = 0.30, 95% CI (0.08–0.53), *p* = 0.009] or adjusted by lifestyle [β = 0.25, 95% CI (0.02–0.47), *p* = 0.03]. The association of decreased H4 acetylation of CD14 with no maternal fish consumption observed in girls was driven by the group of non-anthroposophic mothers [β = 0.39, 95% CI (0.2–0.58), *p* < 0.0001]. H4 acetylation levels in placentas were not associated with the maternal use of olive oil as the main cooking fat during pregnancy or the regular intake of butter or butter-based, full-fat margarine on bread (data not shown). Other factors such as maternal age at birth, parity, birth weight, parental smoking, and gestational age (Table 1) were not associated with H4 acetylation levels of *CD14* (data not shown).

The relationship between maternal fish consumption and H4 acetylation levels in placenta could be attributed to ω-3 polyunsaturated fatty acids (n-3 PUFAs) present in fish oil and is in agreement with previous studies suggesting that PUFAs can modify the epigenome [28]. Indeed, we have recently shown that prenatal fish oil supplementation affects acetylation of immune genes such as protein kinase C zeta gene (*PRKCZ*), *IL13*, and the T-box 21 gene (*TBX21*) [11].

Observations of associations in certain lifestyle groups merits further investigation but may be attributable to interactions with other factors present in these lifestyles. Indeed, specific combinations of nutritional factors may affect the outcomes of a given exposure. For instance, the beneficial effects of grape polyphenols are ameliorated by the presence of high-fat diets [29]. In this study, the maternal intake of olive oil contributed to more pronounced effects on the H3 acetylation levels in the placentas of anthroposophic families in comparison with non-anthroposophic families (Figure 2). Moreover, H4 acetylation levels at the CD14 promoter were similar in all mothers, irrespective of their lifestyle, provided that they had consumed fish during pregnancy; however, for mothers who did not consume fish, the difference in H4 acetylation was significant only in the group of non-anthroposophic mothers (Figure 3). We hypothesize that other exposures in the anthroposophic environment “compensated” for the lack of maternal intake of fish and led to detectable acetylation levels even if the mother did not consume fish (Figure 3). Our sample size precluded combined effects analyses, such as “olive oil + butter” or “fish + butter” on H3 and H4 acetylation levels. A vegetarian diet was also a factor that may have influenced these results but the sample size (Table 1) precluded any formal analysis. Accordingly, future interaction studies are needed to identify factors in the anthroposophic lifestyle that may be implicated in modifying histone acetylation levels.

The association detected in placentas of female offspring is in agreement with recent experimental models showing that the maternal diet exerts sex-specific effects on fetuses and placentas [18] and that sexual dimorphism does exist in the placental response to the maternal environment [30]. A high-fat diet during gestation triggers sex-specific epigenetic alterations in CpG sites (i.e. regions of DNA where a cytosine nucleotide is directly followed by a guanine nucleotide in the linear 5’→3’ order) throughout the genome, together with the deregulation of clusters of imprinted genes [31].

Because the specimens analyzed in this study comprise the entire thickness of the placenta, the cell heterogeneity did not allow any interpretation as to which cells the H3 and H4 acetylation changes could be ascribed. In addition, the analysis of H3 and H4 pan-acetylation levels in each of the genes did not allow us to discriminate individual marks, which underlie the associations detected in this study. Therefore, the association signals detected here require additional fine-mapping with higher resolution methods to solve histone landscapes. Furthermore, elevated H3 and H4 acetylation levels are presumed to promote increased mRNA expression. However, several studies indicate that functional effects of histone acetylation affect the expression of other genomic regions but not the directly underlying gene. For example, a recent study by Kelly et al. indicated that only 10% of the genes showing differential histone acetylation demonstrated differential gene expression [32]. It is also well known that transcriptional activation at many promoters is not associated with increased histone acetylation [33]. Therefore, measuring mRNA expression in genes that show differential histone acetylation in placenta would have been very informative on the potential functional effects of the measured acetylation differences. Unfortunately, the design of our study precluded robust analyses of this type. The manner in which the H3 and H4 acetylation differences found in the placenta are reflected at the gene expression level merits further research using the data acquired from this study. 

In conclusion, this study provides the first empirical evidence that maternal dietary exposure to olive oil and fish are associated with histone acetylation levels in human placenta. How these epigenetic changes contribute to immune maturation and immune development in fetal tissues merits additional study. An increased understanding of the modifying effects of olive oil intake during pregnancy on acetylation open new preventive avenues and lay the foundation for further mechanistic studies.

## 3. Methods

### 3.1. Study Population—the ALADDIN Cohort

Individuals analyzed in this study belonged to the prospective birth cohort Assessment of Lifestyle and Allergic Disease During INfancy (ALADDIN), which consists of families recruited between September 2004 and November 2007 at anthroposophic and conventional maternal health care centers in the Stockholm area, Sweden. Classification of the participating families into an anthroposophic, partly anthroposophic, or non-anthroposophic lifestyle is described in detail elsewhere [21]. Inclusion criteria for the present study were no severe illness before or during pregnancy, ≥36 weeks of gestation, and availability of snap frozen placenta specimens stored at −80 °C; these criteria resulted in 173 placentas. The study was conducted in accordance with the Declaration of Helsinki and was approved by the Regional Ethical Review Board in Stockholm (project Dnr 2010/1811-32; approval date: 15 November 2010). All parents gave their written informed consent for inclusion.

### 3.2. Collection of Placenta Specimens and Histopathology Examination

The placentas (*n* = 173) were collected by midwifes directly after birth, put on ice, and sent to the laboratory at the Karolinska University Hospital (Solna, Sweden). Because deliveries occurred at different hospitals and in diverse homes, transportation time was anticipated to vary. As such, we recorded the time from delivery until proper storage in the laboratory for each placenta. There were no significant differences in the placentas stored on ice between the non-anthroposophic group, median 23.5 h (range 5–73 h, *n* = 43) and the fused anthroposophic + partly anthroposophic group, median 19.3 h (range 2–91 h, *n* = 128; *p* = 0.092, Mann-Whitney U test). From each placenta, a cross-sectional sample about 0.5 cm thick, 1.5 cm wide, and spanning the whole thickness of the placenta was cut near the umbilical cord; each sample was quickly washed two times in phosphate-buffered saline (PBS) to remove as much blood as possible, snap frozen on dry ice, and stored at −80 °C.

### 3.3. Isolation of Chromatin, Chromatin Immunoprecipitation, and Quantitative Polymerase Chain Reaction (qPCR)

A subsection spanning the thickness of the placenta sample was first kept for 8 min in 1 mL 1% paraformaldehyde (Sigma-Aldrich, Munich, Germany) at room temperature (RT). Next, the sample was centrifuged for 5 min at 7870 *g* at RT, incubated with 1 mL 0.25% trypsin ethylenediaminetetraacetic acid (Thermo Fisher Scientific, Waltham, MA, USA) for 1 h at RT, and then centrifuged again for 5 min at 7870 g at RT. The supernatant was discarded and the tissue components were incubated with 0.1% collagenase (Roche Diagnostics, Mannheim, Germany) for another hour at RT, and then centrifuged again for 5 min at 7870 *g* at RT. To purify cells from tissue remnants and cell debris, the pellet was then re-suspended in 1 mL phosphate buffered saline (PBS; Sigma-Aldrich) and run through a 0.2 μm sieve. Next, the cells were washed twice with 1 mL PBS. Further steps, including chromatin purification, chromatin immunoprecipitation (ChIP), and quantitative assessment of both H3 and H4 histone acetylation at the gene promoters by polymerase chain reaction (PCR), were conducted as validated and described before [34] using the PCR primers presented in Table 3.

Three-level strategy of PCR data normalization was applied. First, percent enrichment to the input control was calculated for each target locus and a positive control gene encoding ribosomal protein L32 (*RPL32*), separately for mock (IgG), and H3 and H4 antibodies. Then, locus-specific percent enrichment to the input control obtained for IgG was subtracted from the corresponding values for H3 or H4 antibodies. Such calculated IgG-corrected percent enrichment was divided for each gene into that of *RPL32*, resulting in a relative enrichment value, which was used for subsequent statistical analyses [34,35]. Intra- and interassay coefficients of variation calculated for percent enrichment should not exceed 10% [34]. All samples were processed according to the same standardized protocol, analyzed blinded, and in a randomized order.

### 3.4. Statistics

A comparison of demographic and exposure variables between the study groups were performed by χ^2^ and the Mann-Whitney U test. Given the distribution of the acetylation data, the relationship between exposure factors on histone acetylation levels were first calculated using the Mann-Whitney U test. Subsequently, more detailed analyses, including those with adjustments, were performed by median regression, which generally outperforms standard linear regression in the presence of outliers or asymmetric, possibly heavy-tailed distributions. Regression coefficients with 95% confidence intervals (CIs) were reported. With binary predictors, the regression coefficient represents a difference between medians; with continuous predictors, it is interpreted as the change in the median outcome associated with a unit increase in the predictor. To fit quantile regression models and compute standard errors, we used conventional bootstrap techniques, as implemented by the R package quantreg, version 5.29 (https://cran.rproject.org/web/packages/quantreg/). A value of *p* < 0.05 was considered statistically significant.

## Figures and Tables

**Table 1:** Demographic data of participating families.

Characteristic	Non-Anthroposophic (*N* = 43)	Anthroposophic + Partly Anthroposophic (*N* = 25 + 105)	*p*-Value *
Maternal age (years)	30 (28–33)	31 (27–34)	0.46
Parity			
first	17/43 (40%)	53/128 (41%)	0.97
second	18/43 (42%)	49/128 (38%)	0.81
third or more	8/43 (19%)	26/128 (20%)	0.98
Mother vegetarian diet during pregnancy	2/38 (5%)	22/126 (17%)	0.11
Mother smoking during pregnancy	8/42 (19%)	9/127 (7%)	0.05
Father smoking during pregnancy	11/41 (27%)	31/125 (25%)	0.96
Female offspring	28/43 (65%)	65/130 (50%)	0.12
Birth weight (gram)	3510 (3312–4010)	3568 (3348–3939)	0.64
Gestational age in weeks	39 (38–40)	40 (39–41)	0.01

Continuous variables are presented as median (interquartile range). Categorical variables are presented as *n*/*N* = yes/total number (%). * Chi-square test for comparing categorical variables and Mann-Whitney U test for continuous variables.

**Table 2:** Association between olive oil consumption and H3 acetylation levels in placenta in anthroposophic and partly anthroposophic families (*n* = 125 *).

Predictor: Olive Oil (Yes)	β (95% CI), *p*-Value (Crude)	β (95% CI), *p*-Value (Adjusted by Offspring Gender)
H3 *FOXP3*	0.26 (0.08–0.43), *p* = 0.004	0.21 (0.01–0.41), *p* = 0.03
H3 *IL10RA*	0.31 (0.09–0.54), *p* = 0.008	0.31 (0.08–0.54), *p* = 0.008
H3 *IL7R*	0.36 (0.10–0.62), *p* = 0.007	0.36 (0.10–0.61), *p* = 0.006

* Families with complete data on maternal dietary use of olive oil (see Figure 2). CI denotes confidence interval.

**Table 3:** Primers used for quantitative assessment of H3 or H4 histone acetylation.

Gene	Forward Primer	Reverse Primer
*FOXP3*	ATCGTGAGGATGGATGCATTAATA	CCACTGGGAAGGTCCCTAGC
*IL10RA*	GCAACTACCTCCTCCCCATT	GCCTTCGGATCAAAGTGGTC
*IL7R*	AACCCCGTCTCCACTGAAAA	GAGTCTTGCTTTGTTGCCCA
*CD14*	ATCAGGGTTCACAGAGGA	GACCCCAAGACCCTACAC
*RPL32*	GGAAGTGCTTGCCTTTTTCC	GGATTGCCACGGATTAACAC
